# Machine learning for optimizing daily COVID-19 vaccine dissemination to combat the pandemic

**DOI:** 10.1007/s12553-022-00712-4

**Published:** 2022-11-10

**Authors:** David Opeoluwa Oyewola, Emmanuel Gbenga Dada, Sanjay Misra

**Affiliations:** 1 Department of Mathematics and Computer Science, Federal University Kashere, Gombe, Nigeria; 2 Department of Mathematical Sciences, University of Maiduguri, Maiduguri, Nigeria; 3 Department of Computer Science and Communication, Østfold University College, Halden, Norway

**Keywords:** COVID-19, Vaccination, Machine learning, Gaussian process (GAUSS), Elastic net (ENET), Spikes and slab (SPIKES)

## Abstract

**Introduction:**

Vaccines are the most important instrument for bringing the pandemic to a close and saving lives and helping to reduce the risks of infection. It is important that everyone has equal access to immunizations that are both safe and effective. There is no one who is safe until everyone gets vaccinated. COVID-19 vaccinations are a game-changer in the fight against diseases. In addition to examining attitudes toward these vaccines in Africa, Asia, Oceania, Europe, North America, and South America, the purpose of this paper is to predict the acceptability of COVID-19 vaccines and study their predictors.

**Materials and methods:**

Kaggle datasets are used to estimate the prediction outcomes of the daily COVID-19 vaccination to prevent a pandemic. The Kaggle data sets are classified into training and testing datasets. The training dataset is comprised of COVID-19 daily data from the 13th of December 2020 to the 13th of June 2021, while the testing dataset is comprised of COVID-19 daily data from the 14th of June 2021 to the 14th of October 2021. For the prediction of daily COVID-19 vaccination, four well-known machine learning algorithms were described and used in this study: CUBIST, Gaussian Process (GAUSS), Elastic Net (ENET), Spikes, and Slab (SPIKES).

**Results:**

Among the models considered in this paper, CUBIST has the best prediction accuracy in terms of Mean Absolute Scaled Error (MASE) of 9.7368 for Asia, 2.8901 for America, 13.2169 for Oceania, and 3.9510 for South America respectively.

**Conclusion:**

This research shows that machine learning can be of great benefit for optimizing daily immunization of citizens across the globe. And if used properly, it can help decision makers and health administrators to comprehend immunization rates and create strategies to enhance them.

## Introduction

The World Health Organization (WHO) declared the coronavirus outbreak in Wuhan, China, in December 2019 as COVID-19, designating 2020 as the year of global disaster [1]. Since the first fatality from the disease was recorded in January 2020, before vaccination began in the United Kingdom on December 8th, the number of confirmed and death cases has progressively increased (UK). The virus spreads from person to person, resulting in a worldwide epidemic. COVID-19 is a virus that causes mild symptoms in persons of various ages [2, 3].

While data indicates that two categories of persons are much more likely to be infected by this serious virus than some others: the aged (over 60 years old) and those who are habitually sick as a result of congestive heart failure, diabetes, acute pulmonary, and cancer problems. The number of confirmed cases is dropping fast in nations where vaccines have started, such as the United States and the United Kingdom, whereas it is still rising or constantly changing in places where immunizations have begun late or not at all. Because the death toll from COVID-19 has been steadily rising over the world [4], many countries have been compelled to take more drastic measures, such as masking, social distancing, isolation, and restriction. The creation of the COVID-19 vaccines as soon as possible has become a worldwide concern. In 2022, sufficient COVID-19 vaccines are expected to be manufactured to immunize a minimum of 70 percent [5]. Based on the most recent research, there is a quantity that might halt the global epidemic years earlier than projected if the vaccines are well circulated.

Due to their great efficiency, machine learning (ML) techniques are now widely employed in a wide range of computer applications [6, 7]. Empirical methods such as data mining and machine learning, for example, might help. Machine learning classification algorithms are data processing methods that make it easier to create closed-form mathematical models [8, 9]. Machine learning classification algorithms are data processing techniques that make the building of analytical models easier. Data mining is used to create rules from massive amounts of data. Machine learning is crucial in today’s environment, especially in healthcare. Machine learning techniques are being used to help with hospital system restructuring, infectious disease detection and treatment, and medical treatment [10–13]. In addition, machine learning models may be used to give intelligent solutions for analyzing vast amounts of data.

By building rapid and effective algorithms and models based on actual observations of the process for real-time data processing, machine learning approaches are able to give outstanding outputs and analysis. As a result, more information must be provided to medical workers so that they may make educated judgments regarding medical diagnosis and treatment approaches while also being aware of the potential consequences and costs for everyone [14]. The ability of machine learning to generate massive datasets beyond human competence is critical in healthcare. Following that, machine learning reliably transforms data analysis into the knowledge of the disease and its symptoms, as well as the necessity for therapy. This will help healthcare workers plan and deliver treatment, resulting in improved results, fewer healthcare costs, and more patient satisfaction [15, 16].

The importance of ML in healthcare is its proficiency in generating large datasets over and above human capacity. Afterward, ML dependably transforms the analysis of that data into awareness of the disease and its symptoms, and the need for treatment. This will aid healthcare providers in planning and delivering care, resulting in improved results, lower healthcare costs, and more patient satisfaction [15, 16].

To optimize the daily COVID-19 vaccine so as to fight against the pandemic, we obtained data from Kaggle and analyzed it using machine learning algorithms [17]. ML algorithms are used for extracting useful information from data and building a reliable predictive model from it. Machine learning algorithms [18–21] are used in this study to improve vaccination and determine which is the most successful in terms of consistency and accuracy. The objective of this paper is to apply predict about the acceptability of COVID-19 vaccines and study their predictors.

This research contribution is summarized as follows:

- Machine learning algorithms that are capable of effectively predicting COVID-19 vaccine daily dissemination are presented.
- Novel systems that can predict the trends of COVID-19 vaccination for each continent in the world are proposed.
- A new model for optimal predicting of COVID-19 vaccination is presented.
- A novel system with increased accuracy of COVID-19 vaccination prediction is proposed.

The remainder of this work is structured as follows: Section two discusses relevant work, section three discusses methodology, part four discusses findings and discussion, and the conclusion is presented in section five.

## Related works

Kim [22] used two statistical models and a deep learning (DL) model and (LSTM-DNN) to model and forecast daily verified coronavirus cases (COVID-19). The stacked long short-term memory deep neural network was employed in conjunction with the autoregressive integrated moving average (ARIMA), the generalized autoregressive conditional heteroscedasticity (GARCH), and the autoregressive integrated moving average (ARIMA). The experiment uses ten datasets provided by the WHO. Simulation results reveal that performance is based on the dates and vaccinations utilized in the data. It also reveals that the proposed LSTM-DNN prediction is superior to the two statistical models. According to the experimental data, LSTM-DNN significantly improves mean absolute error when compared to ARIMA and GARCH. ARIMA and GARCH yield different outcomes based on the dataset. The achieved results could serve as a benchmark for the COVID-19 daily confirmed cases' performance limits and prediction accuracy. The shortcoming of the work is that data collected after February 2021 was not included in the analysis. Furthermore, the accuracy of the proposed model is quite poor.

Cheong et al. [23] used machine learning techniques to examine socioeconomic data from a range of online sources, including the US CDC and the US Census Bureau COVID-19 Site. Using XGBoost and socioeconomic data, a machine learning study was done. With a 62 percent accuracy rate, the suggested model accurately predicted COVID-19 vaccination uptake in US countries. It was also observed that the most important socioeconomic determinants determining vaccine uptake in the United States include region, education, ethnicity, income, and home internet access. Finally, the algorithm generated a themed map depicting low and high vaccination rates, which health care officials might utilize in future pandemics to visualize and prioritize low vaccination zones as well as organize specific vaccine programs. The experiment had a flaw in that the dataset only included data from the United States, resulting in poor prediction accuracy.

Machine learning techniques were used by Abdulkareem et al. [24] to assess the progress of COVID-19 immunization throughout the world. The findings of the article indicated which method is superior for which dataset. To assess and generate findings, four output classification strategies were used: Decision Tree (DT), K-nearest neighbors (KNN), Random Tree (RT), and Naive Bayes (NB), with ML models on Weka. According to the research, DT outperforms other algorithms in terms of speed and precision. The experiment performance was not compared with other machine learning algorithms utilizing the same dataset, which is a flaw in the work. Moreover, the performance of many of the ML models used except for the decision tree is poor.

Fernandes et al. [25] used machine learning approaches to predict COVID-19 vaccination and the factors that influence vaccines. The study’s major purpose was to look at people’s intentions to be vaccinated and to vaccinate their children. They also wanted to know how their decision was impacted by personal qualities, emotional concerns, and the lockdown scenario. As a result, during the Portugal shutdown, the authors conducted an online survey (15 January 2021 until 14 March 2021). According to the data, 63% of the 649 participants said they were highly likely to obtain the vaccination and 60% said they would vaccinate their children. The trials employed linear regression models, which explained 65 percent of the variation in individual immunization and 56 percent of the variance in group immunization. The overall ideas and attitudes about the COVID-19 vaccine were revealed to be the most important drivers of vaccination intention. Furthermore, the recommended artificial neural network (ANN) model achieved a vaccination intention prediction accuracy of 85%. The drawback of the work is the prediction accuracy is low. Also, the work did not cover data obtained after 14 March 2021.

To evaluate the general predilection to the COVID-19 vaccination, Zaidi et al. [26] used five models. A voting classifier was used to determine the accuracy of all the classifiers at the completion of their research. According to the data, a Support Vector Machine (SVM) generates the best predictions, whereas an Artificial Neural Network (ANN) produces the worst predictions for individual capacity to be vaccinated with the COVID-19 vaccine. The proposed technique has an overall accuracy of 89.9% for the random dataset and 45.7 percent for the date-wise dataset when employing the voting classifier. As a result, the results reveal that the proposed prediction technique is a valid and promising method for predicting future COVID-19 vaccination trends. The suggested work has a flaw in that the total forecast accuracy is low.

Using previously available data, Davahli et al. [27] created sequence-learning models to estimate the behavior of the COVID-19 pandemic across the United States (US). They classified US states based on their resemblance to previously reported COVID-19 behavior to avoid training the models for all states. The researchers employed an unsupervised self-organizing map to divide all states in the United States into four groups based on the similarity of their effective reproduction numbers. They developed deterministic and stochastic long short-term memory (LSTM) and mixed density network (MDN) models after selecting a leading state (the state with the earliest recorded occurrences) in each group. Data was added into the model from each leading state, which was then compared to a baseline linear regression model to predict future outcomes. They investigated the effects of removing periodicity and patterns from a dataset of non-stationary COVID-19 events on prediction. Alternative prediction strategies beat the deterministic LSTM model trained on the COVID-19 ideal reproduction numbers, according to their findings. The work shortcoming is that the data used is exclusively from the United States. Furthermore, the authors did not take into account the interaction of states. Finally, the study data set is limited to only three months from August 26, 2020, to November 26, 2020. Table 1 summarized the list of all related works.

**Table 1:** List of all the summarized works

**Ref.**	**Proposed Technique**	**Contributions**	**Research Gap**
Kim [22]	Statistical models and deep learning (DL) model (LSTM-DNN)	LSTM-DNN achieved better performance when compared to ARIMA and GARCH’s root mean squared error.	Data collected after February 2021 was not included in the analysis. Also, the accuracy of the proposed model is quite poor.
Cheong et al. [23]	Machine Learning	Discovered the most important socioeconomic factors in predicting vaccination uptake in US.	The dataset employed in the work is limited to the United States, and the prediction accuracy is relatively poor.
Abdulkareem et al. [24]	Decision Tree (DT), K-nearest neighbors (KNN), Random Tree (RT), and Naive Bayes (NB)	In terms of time and accuracy, this was an excellent performance.	The results of the ML models employed in the experiment were not compared to those of high-performing machine learning algorithms utilizing the same dataset. Poor performance of many of the ML models used except for decision tree.
Fernandes et al. [25]	Machine Learning	ANN model achieved vaccination intention prediction accuracy of 85%.	Prediction accuracy is low. Also, the work did not cover data obtained after 14 March 2021.
Zaidi et al. [26]	Machine Learning	Overall accuracy of 89.9% for SVM.	Total prediction accuracy is low.
Davahli et al. [27]	Deterministic and stochastic LSTM and MDN	Alternative prediction techniques were surpassed by a deterministic LSTM model trained on the COVID-19 optimal reproduction numbers.	The dataset that was used was only for the United States. The influence of states on one another was not considered by the authors. Dataset used for the work is limited to just three months data (August 26, 2020 to November 26, 2020).

Motivated by gaps and the benefit mentioned above, Elastic Net (ENET), CUBIST, Gaussian Process (GAUSS), and Spikes and Slab (SPIKES) methods are developed for the prediction of COVID-19 vaccination in Africa, Asia, Europe, South America, North America, and Oceania. To the best of our knowledge, this is the first-time prediction of the COVID-19 vaccine in combating COVID-19 diseases in the continents of the world is explored.

## Methodology

### Dataset

Kaggle datasets [37] were utilized to evaluate the prediction outcomes of the daily COVID-19 vaccination to decrease pandemic risk. The dataset consists of all the countries that have been vaccinated, fully vaccinated with COVID-19, the types of vaccines used and the date vaccinated. Training datasets and testing datasets are the two types of data sets utilized in this study. COVID-19 daily data from the 13th of December 2020 to the 13th of June, 2021 make up the training dataset, whereas COVID-19 daily data from the 14th of June, 2021 to the 14th of October, 2021 make up the testing dataset. Table 2 displayed the attributes of COVID-19 vaccination dataset which consists of country, iso_code, date, total_vaccinations, people_vaccinated, people_fullt_vaccinated, daily_vaccination_raw, daily_vaccinations, total_vaccinations_per_hundred, people_vaccinated_hundred, people_fully_vaccinated_per_hundred, daily_vaccinations_per_million and vaccines.

**Table 2:** Attributes of COVID-19 Vaccination dataset

**Terms**	**Data Type**
Country	Nominal
iso_code	Nominal
Date	Ordinal
total_vaccinations	Continuous
people_vaccinated	Continuous
people_fully_vaccinated	Continuous
daily_vaccinations_raw	Continuous
daily_vaccinations	Continuous
total_vaccinations_per_hundred	Continuous
people_vaccinated_per_hundred	Continuous
people_fully_vaccinated_per_hundred	Continuous
daily_vaccinations_per_million	Continuous
Vaccines	Categorical

### Elastic net (ENET)

Elastic Net (ENET) is a penalized linear regression model that incorporates both the L1 and L2 penalties. Combining the L1-norm (lasso) and L2-norm (ridge) penalties, ENET decreases the regression coefficients. ENET arose from criticism of LASSO (Least Absolute Shrinkage and Selection Operator), a variable selection algorithm that is excessively dependent on data and hence unstable [28]. To obtain the best of both techniques is to mix the penalties of ridge regression and lasso [29]. ENET mathematical equations are as follows:

1 $${E}_{enet}\left(\widehat{\beta }\right)=\frac{\sum{i=1}^{n}{\left({y}_{i}-{x}_{i}\widehat{\beta }\right)}^{2}}{2n}+\gamma \left(\frac{1-\alpha }{2}\sum\nolimits_{j=1}^{m}{{\widehat{\beta }}_{j}}^{2}+\alpha \sum\nolimits_{j=1}^{m}\left|\widehat{{\beta }_{j}}\right|\right)$$

where $\alpha$ is the mixing parameter between ridge ($\alpha =0$) and lasso $(\alpha =1)$, $n$ is the observation of the response variable, ${y}_{i},$ with a linear combination of $m$ predictor variables, ${x}_{i},$ $\gamma$ is the regularization penalty, $\beta$ is the regression coefficient.

### CUBIST

Cubist is a rule-based model derived from Quinlan's M5 model tree. Linear regression models are embedded in the terminal leaves of a tree. The predictors used in earlier splits have been utilized to create these models. At each branch of the tree, there are also intermediate linear models. At the tree's terminal node, a prediction is created using the linear regression model, but it is "smoothed" by taking into consideration the preceding node's prediction (which also occurs recursively up the tree). The tree is simplified to a collection of rules, which are originally pathways from top to bottom [30]. CUBIST has the following mathematical equation:

2 $${C}_{cubist}=\left(1-a\right)\times \rho \left(p\right)+a\times \rho (c)$$

where $\rho (c)$ is the current model forecast and $\rho \left(p\right)$ is the parent model prediction positioned above it in the tree.

### Gaussian process (GAUSS)

The Gaussian Processes (GAUSS) model is a probabilistic machine learning framework that is often used for regression and classification issues [31]. The GAUSS model may make predictions based on past data and provide confidence ranges for those predictions. The Gaussian processes model [32] is an approach developed by scientist and statistician. The following are the GAUSS mathematical procedures:

The following is a multivariate Gaussian regression function:

3 $$P\left(f|X\right)=\aleph (f|\mu ,k)$$

The $f$ and ${f}_{*}$ joint distribution is given as

4 $$\left[\begin{array}{c}f\\ {f}_{*}\end{array}\right]\sim \aleph \left(\left[\begin{array}{c}m\left(X\right)\\ m\left({X}_{*}\right)\end{array}\right],\left[\begin{array}{cc}k& {k}_{*}\\ {k}_{*}^{T}& {k}_{**}\end{array}\right]\right)$$

The following is the combined distribution of observed values and function values at new testing points:

5 $$\left(\begin{array}{c}y\\ {f}_{*}\end{array}\right)\sim \aleph \left(0,\left[\begin{array}{cc}k+{\sigma }^{2}\mathrm{I}& {k}_{*}\\ {k}_{*}^{T}& {k}_{**}\end{array}\right]\right)$$

Predictive equations for Gaussian processes regression may be found by determining the conditional distribution:

6 $$\widehat{{f}_{*}}|X,y,{X}_{*}\sim \aleph (\widehat{{f}_{*}},cov\left({f}_{*}\right))$$

Also,

7 $$\widehat{{f}_{*}}\triangleq \left[\widehat{{f}_{*}}|X,y,{X}_{*}\right]={k}_{*}^{T}{\left[k+{\sigma }_{n}^{2}I\right]}^{-1}y$$

8 $$cov\left({f}_{*}\right)={k}_{**}-{k}_{*}^{T}{\left[k+{\sigma }_{n}^{2}I\right]}^{-1}{k}_{*}$$

where $$X=\left[{x}_{1},\dots ,{x}_{n}\right],$$ $$f=\left[f\left({x}_{1}\right),\dots .,f\left({x}_{n}\right)\right],$$ $$\mu =\left[m\left({x}_{1}\right),\dots ,m\left({x}_{n}\right)\right],$$  $${k}_{ij}=k\left({x}_{i},{x}_{j}\right), X$$ is the observed data points, $$m$$ represents the mean function, $$k$$ represents a positive definite kernel function,$$k=k\left(X,X\right),$$  $${k}_{*}=k\left(X,{X}_{*}\right),$$$${k}_{**}=k\left({X}_{*},{X}_{*}\right),$$  $$\left(m\left(X\right),m\left({X}_{*}\right)\right)=0$$.

### Spikes and slab (SPIKES)

Spike and slab regression was alluded by [33] who adopted a Bayesian strategy to subgroup selection in linear regression models. [34, 35] contributed considerably to the development of the technique. The final adjustments to the model were done by [36]. The prior for the regression coefficients utilized in their Bayesian hierarchy was referred to by spike and slab. The mathematical equations of SPIKES is given as:

9 $$(y\left|X,\beta ,{\sigma }^{2}\right)\sim \aleph \left(X\beta ,{\sigma }^{2}{I}_{n}\right)$$

10 $$\left(\beta |\gamma \right)\sim \aleph (0, \omega )$$

### Experimental design of COVID-19 vaccination prediction system

**Fig. 1:**
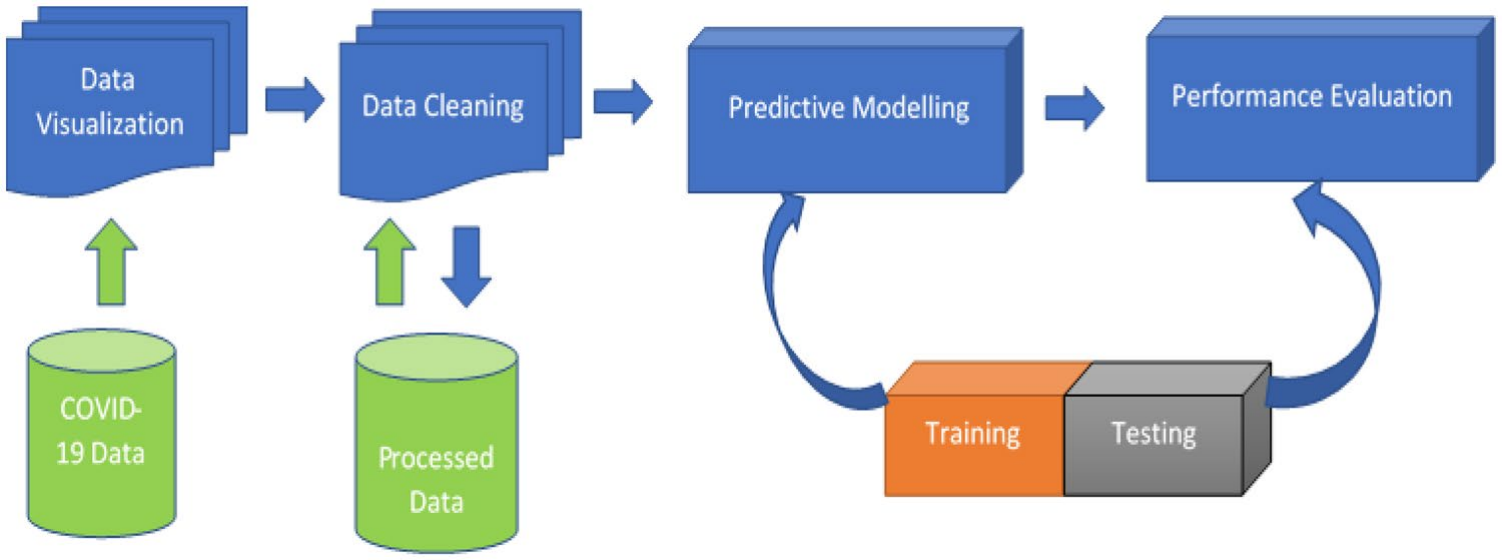
Depicts the block diagram of the proposed COVID-19 vaccination

This paper used four machine learning models for COVID-19 vaccination prediction as depicted in Fig. 1. The proposed architecture uses time-series data preprocessed to extracts spatial features using different machine learning models. The attributes of COVID-19 vaccination dataset obtained from Kaggle comprises of Country, iso_code, Date, total_vaccinations, people_vaccinated, people_fully_vaccinated, daily_vaccinations_raw, daily_vaccinations, total_vaccinations_per_hundred, people_vaccinated_per_hundred, people_fully_vaccinated_per_hundred, daily_vaccinations_per_million, Vaccines. The dataset was partitioned into training set (70%) and test set (30%). Afterward, selected features of the data were modeled using Elastic Net (ENET), CUBIST, Gausian Process (GAUSS), and Spikes and Slab (SPIKES) algorithms. The accuracy of the machine learning is evaluated in the performance evaluation section. Training a model entail selecting appropriate values for each weight and bias from labelled samples. Tuning parameters is one of the most crucial steps in the training of machine learning models. The parameters used to regulate the COVID-19 vaccine training set are all shown in Table 3 and are used to fine-tune the model's performance.

**Table 3:** Training Control Parameters of ENET, CUBIST, GAUSS and SPIKES

**Arguments**	**Parameter**	**Meaning**
Method	The resampling method: repeatedcv	Provides a way to improve the estimated performance of machine learning
Number	10	The number of resampling iterations
Search	random	Describing how the tuning parameter is determined
Repeats	3	Is the number of complete set of folds to compute
verboseIter	TRUE	A logical for printing a training log

### Performance measures

Three metrics are used to evaluate prediction performance of daily COVID-19 vaccination: Mean Absolute Scaled Error (MASE), Relative Absolute Error (RAE), Mean Squared Log Error (MSLE).

MASE is given a:

11 $$\frac{1}{n}\sum\nolimits_{n=1}^{n}(\frac{\left|{y}_{t}^{n}- {\widehat{y}}_{t}^{n}\right|}{\frac{1}{n-m}\sum_{n=m+1}^{n}\left|{y}_{t}^{n}- {y}_{t-m}^{n}\right|})$$

RAE is defined as follows:

12 $$\frac{\sqrt{\sum_{n=1}^{n}{\left({y}_{t}^{n}-{\widehat{y}}_{t}^{n}\right)}^{2}}}{\sqrt{\sum_{n=1}^{n}{{y}_{t}^{n}}^{2}}}$$

MSLE is defined as follows:

13 $$\frac{1}{n}\sum_{n=0}^{n}({\mathrm{log}\left({y}_{t}^{n}+1\right)-\mathrm{log}({\widehat{y}}_{t}^{n}+1))}^{2}$$

where $m$ is the seasonal period, ${y}_{t}^{n}$ is the actual values and predicted values is ${\widehat{y}}_{t}^{n}$.  

## Result and discussion

**Fig. 2:**
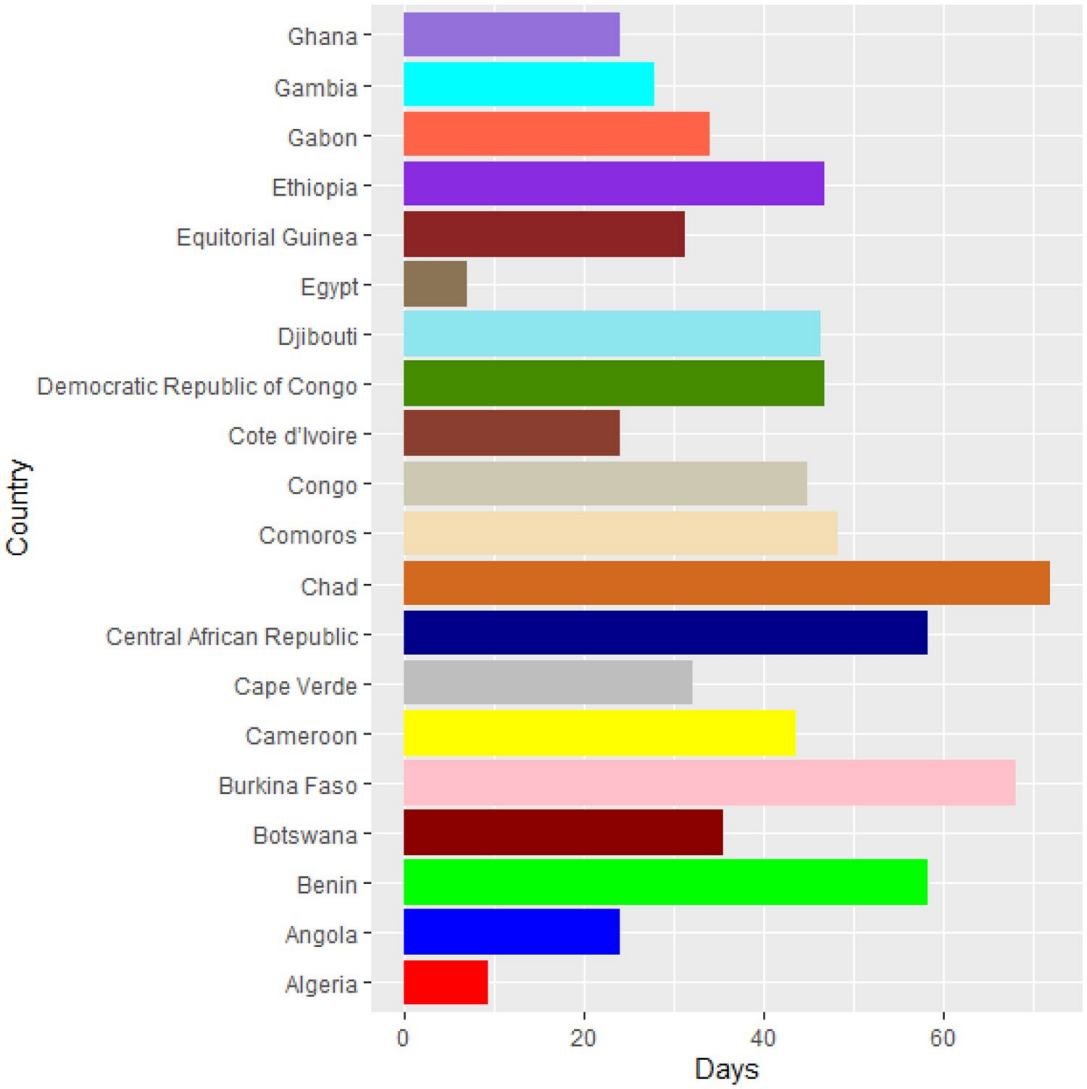
Vaccination against COVID-19 in Africa

**Fig. 3:**
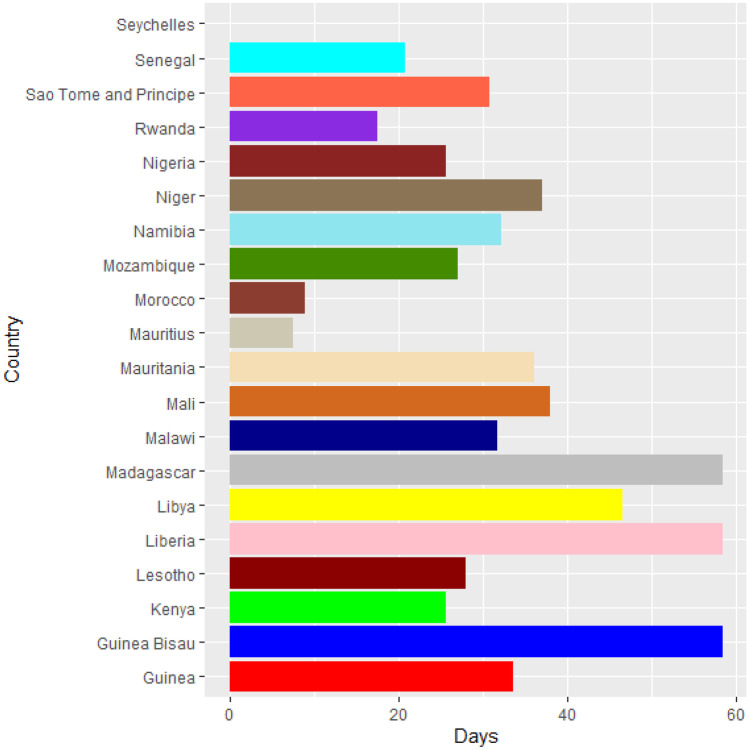
Vaccination against COVID-19 in Africa (Continued)

**Fig. 4:**
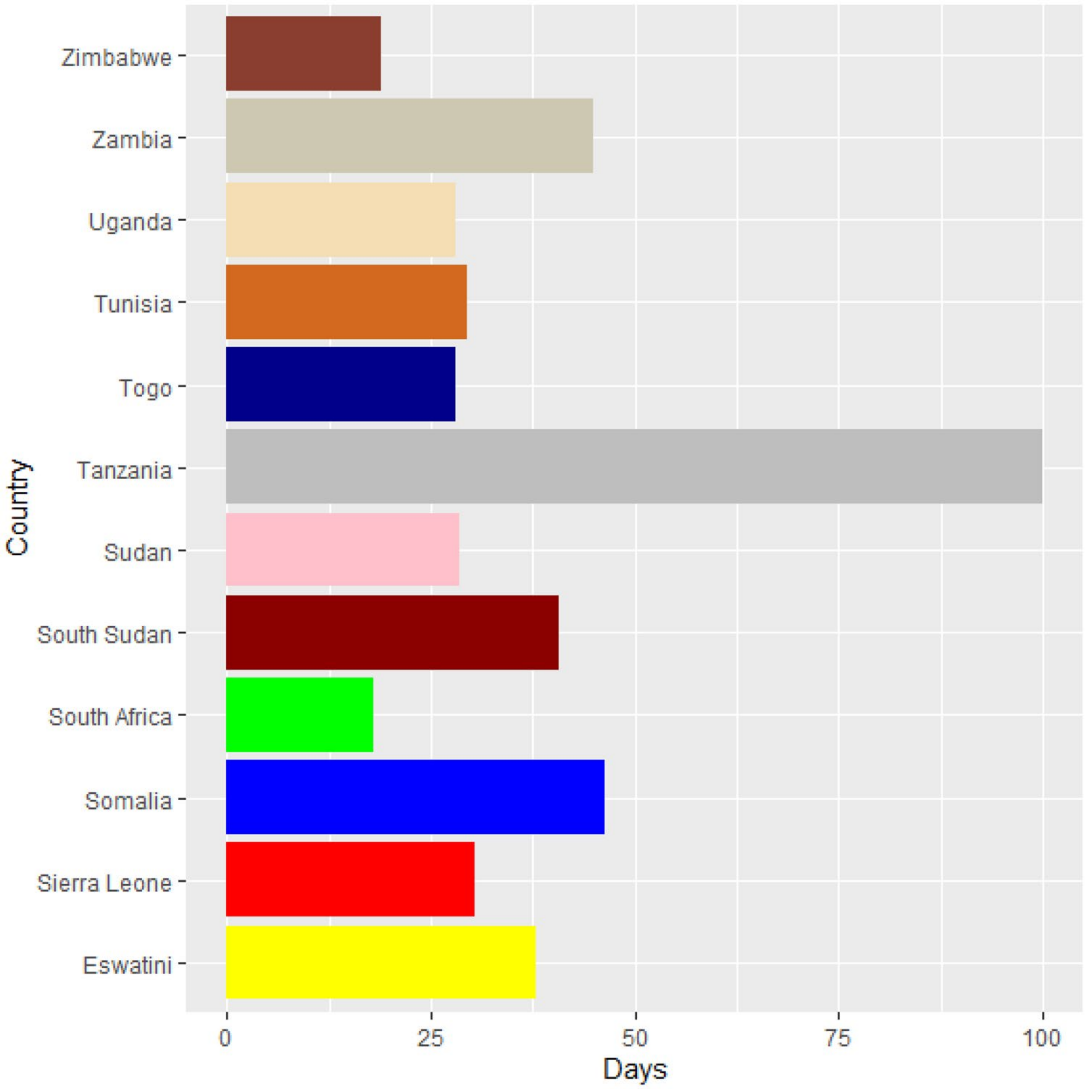
Vaccination against COVID-19 in Africa (Continued)

**Fig. 5:**
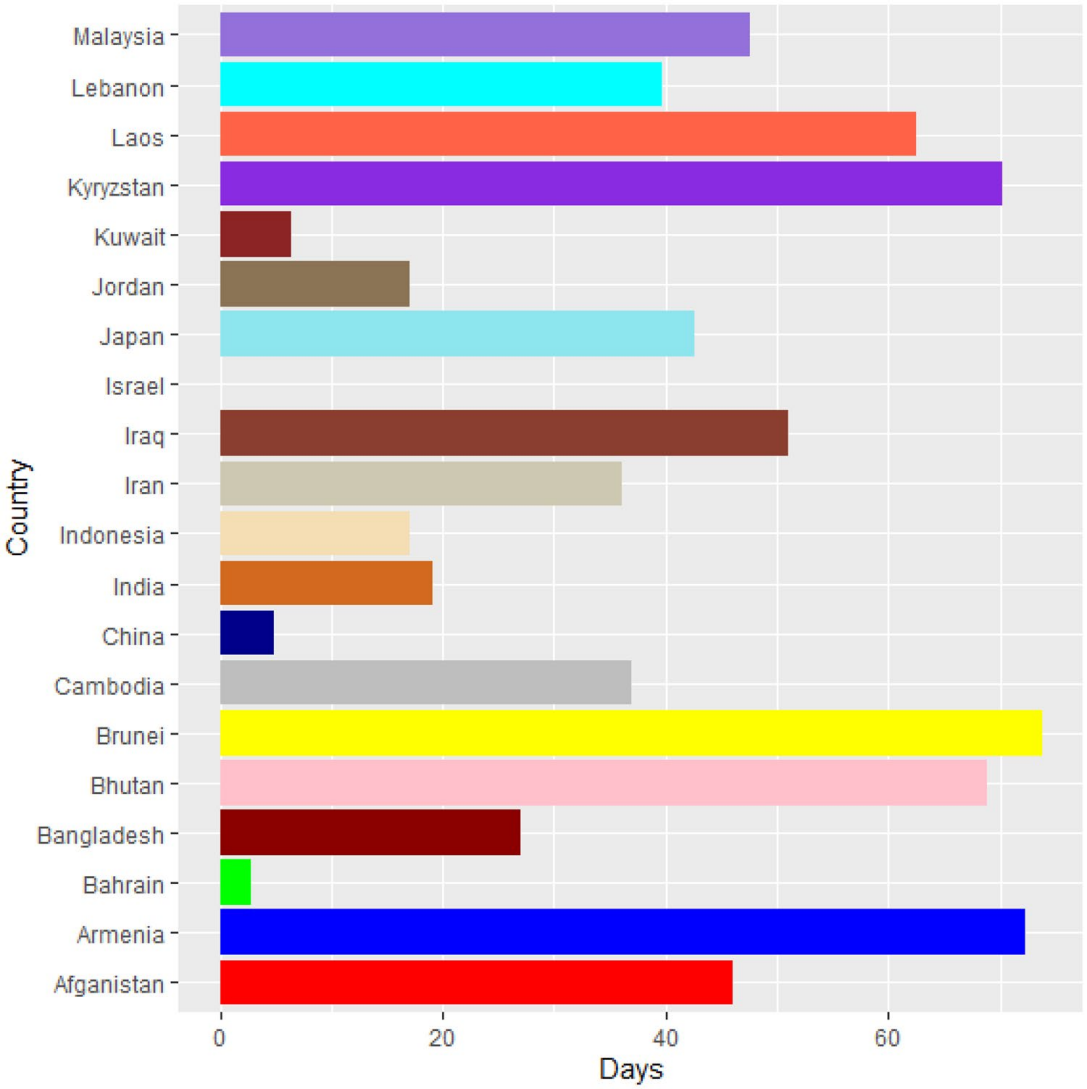
Vaccination against COVID-19 in Asia

**Fig. 6:**
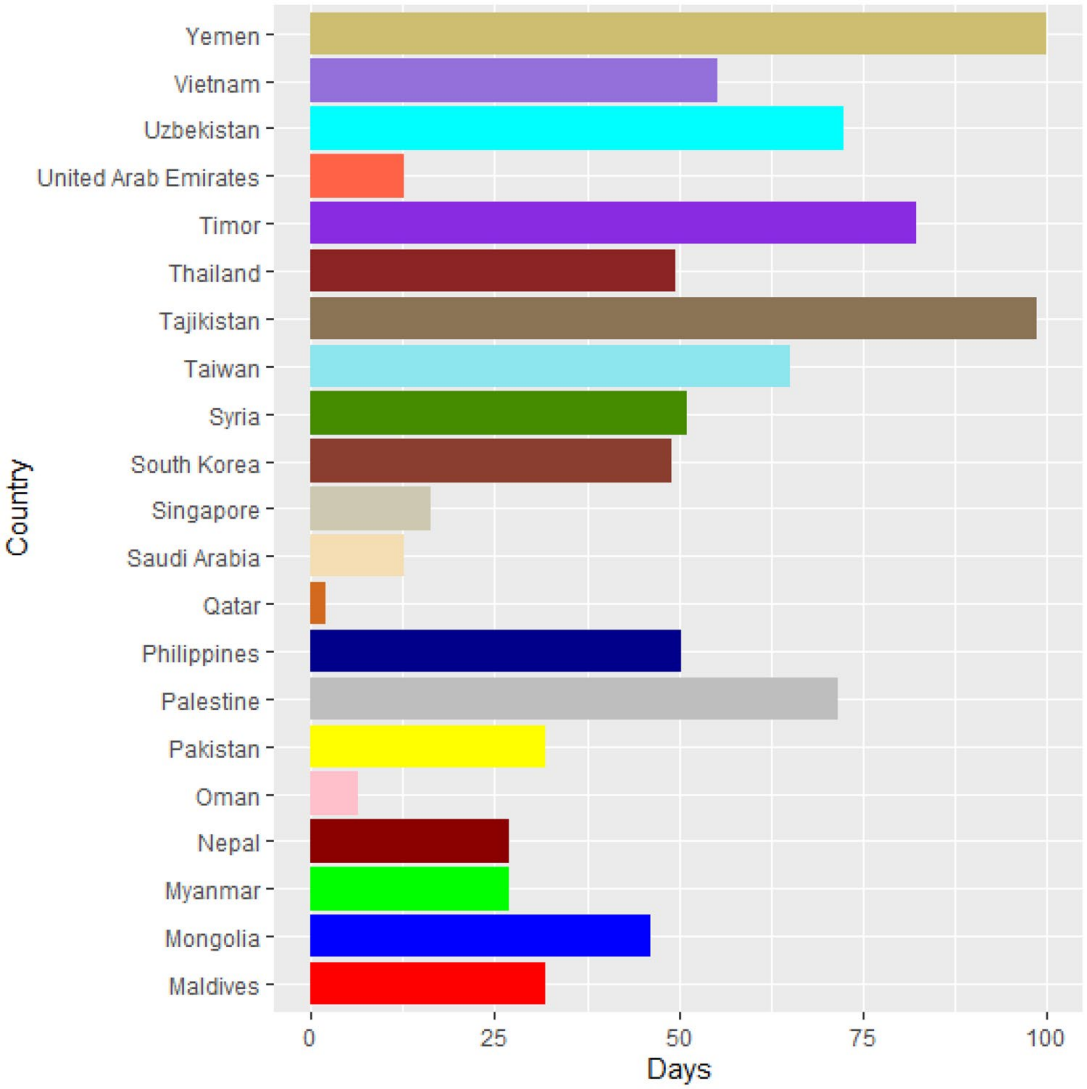
Vaccination against COVID-19 in Asia (Continued)

**Fig. 7:**
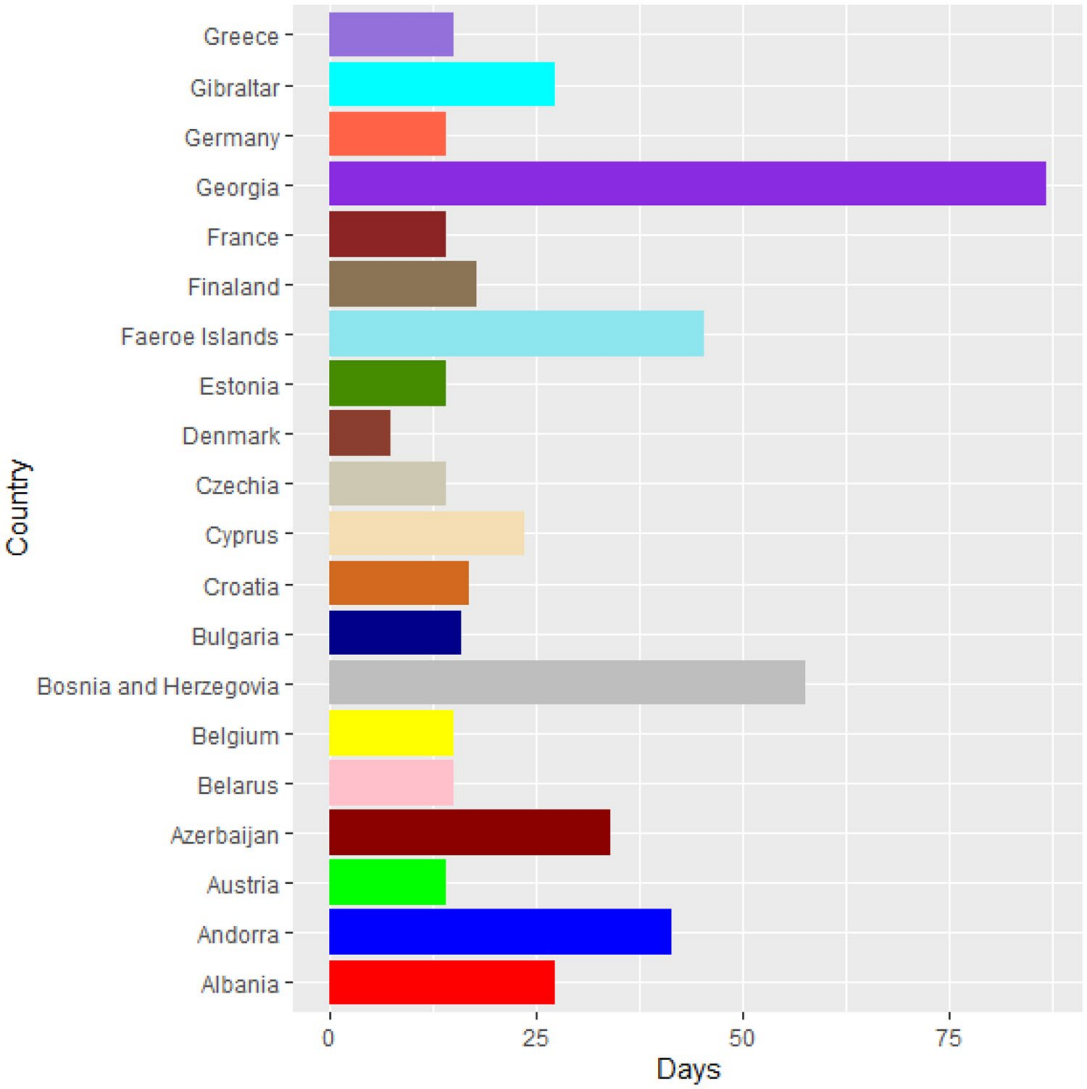
Vaccination against COVID-19 in Europe

**Fig. 8:**
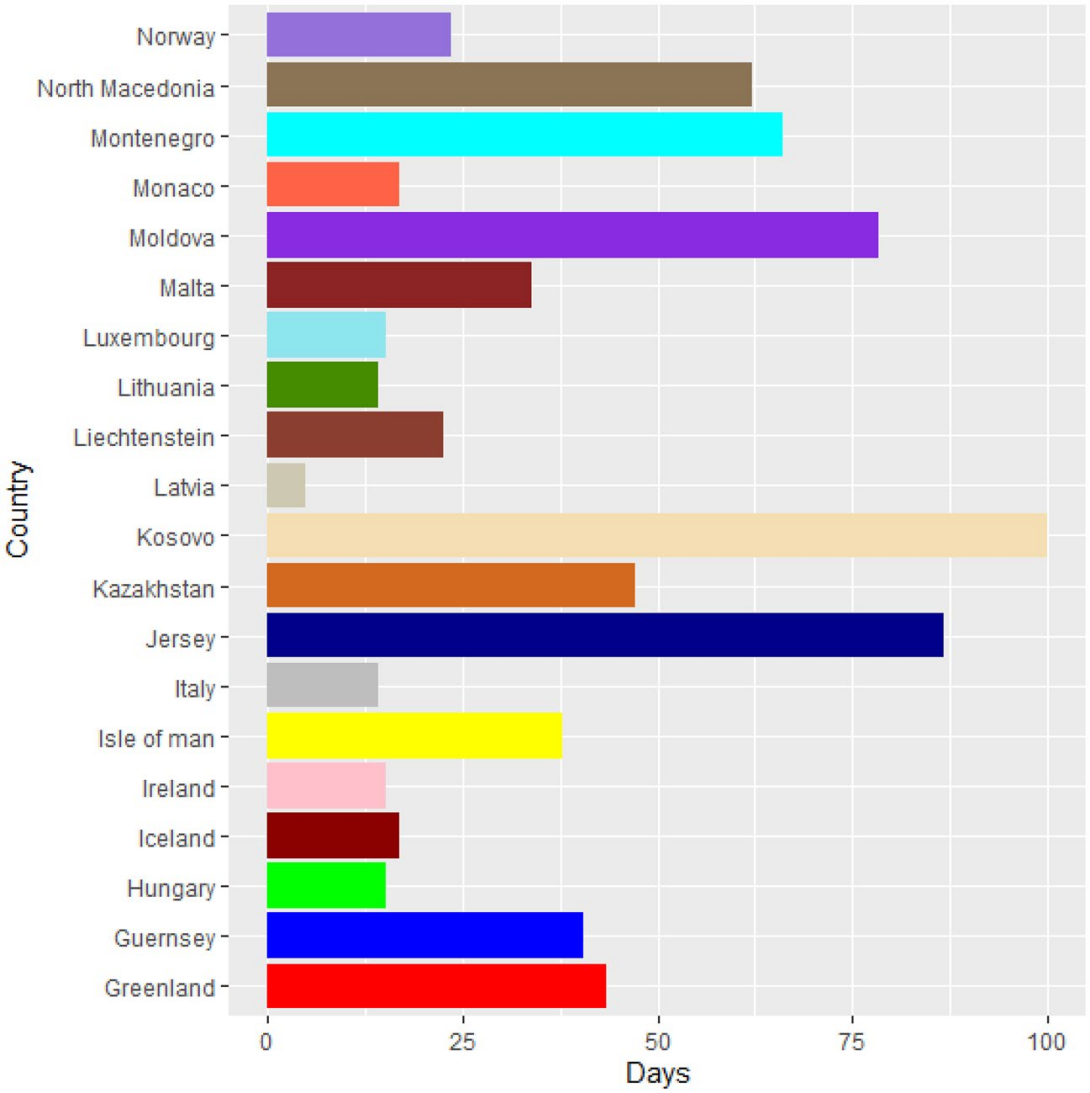
Vaccination against COVID-19 in Europe (Continued)

**Fig. 9:**
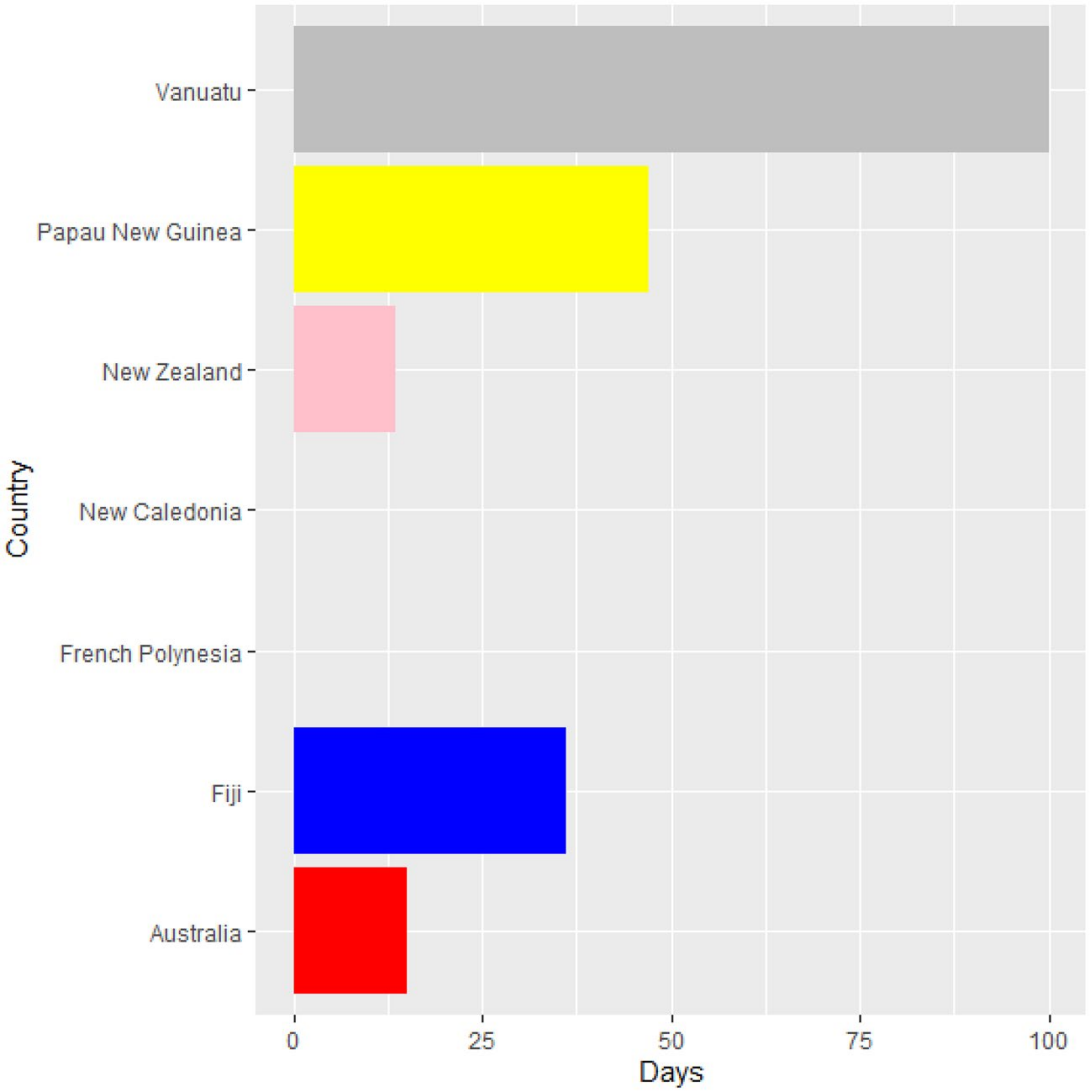
Vaccination against COVID-19 in Oceania

**Fig. 10:**
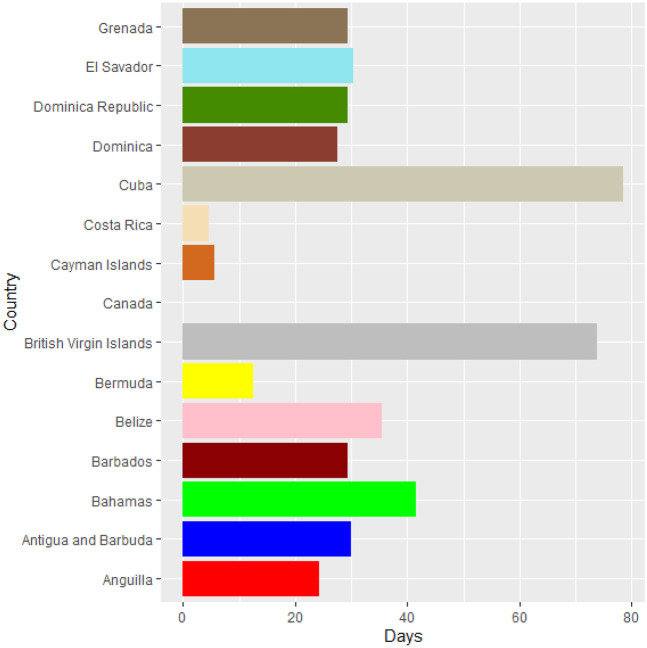
Vaccination against COVID-19 in North America

**Fig. 11:**
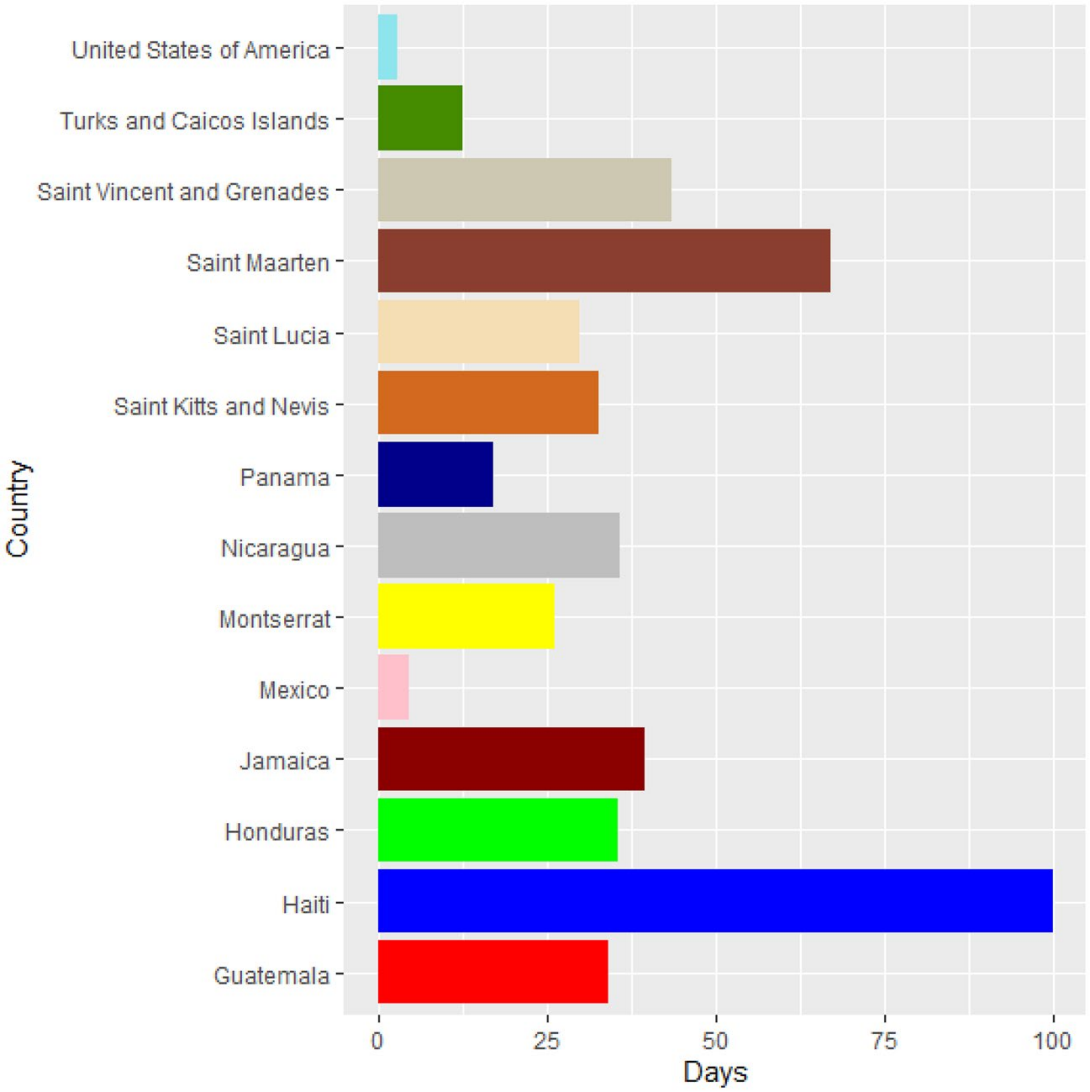
Vaccination against COVID-19 in North America (Continued)

**Fig. 12:**
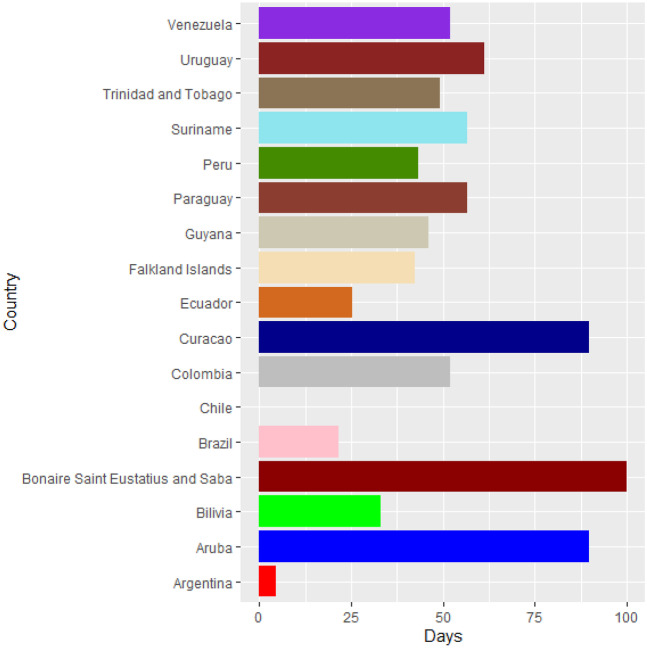
Vaccination against COVID-19 in South America

Vaccines are the most important instrument for bringing the pandemic to an end and saving lives and livelihoods. It is critical that everyone has equal access to immunizations that are both safe and effective. There is no one who is safe until everyone gets vaccinated. In the fight against COVID-19, safe and effective COVID-19 vaccinations are a game-changer. Figures 2–12 is the roll-out of COVID-19 vaccines for each country of the World. Figure 2 consist of twenty countries in Africa that have commences vaccine of its citizen. Egypt and Algeria commenced vaccination of its citizen earlier than other country while Chad and Burkina Faso commenced late in the vaccination chart. The country that first commenced COVID-19 vaccination among them are Seychelles and Mauritius while three countries commenced late and these are Madagascar, Liberia and Guinea Bissau as shown in Fig. 3. Out of the twelve countries in Fig. 4, South Africa and Zimbabwe commenced vaccination earlier than others while Zambia, Tanzania and Somalia commenced vaccination of their citizen late. Out of twenty countries in Asia, China, Kuwait and Bahrain commenced vaccination earlier while Kyryzstan, Brunei and Armenia commenced late in the vaccination of their citizen (as shown in Fig. 5). Out of twenty-one countries in Fig. 6, Qatar and Oman commenced vaccination earlier than others while Yemen and Tajikistan commenced late in the vaccination of its citizen. Almost all the countries in Fig. 7 commenced vaccination of its citizen earlier except Georgia, Faeroe Islands and Bosnia and Herzegovia that commenced vaccination late. Out of twenty-one of this countries Moldova, Kosovo and Jersey commenced vaccination of its citizen late than other countries (as shown in Fig. 8. Figure 9 consists of eight countries in Oceania which include Vanuatu, Papua New Guinea, New Zealand, New Caledonia, French Polynesia, Fiji and Australia. Out of the eight countries in Oceania, two countries commenced vaccination earlier and these are New Caledonia, French Polynesia. Figures 10, 11, 12 consists of countries in South America that commences vaccination. It was observed Canada, USA, Mexico, Chile and Argentina commenced vaccination earlier.

**Fig. 13:**
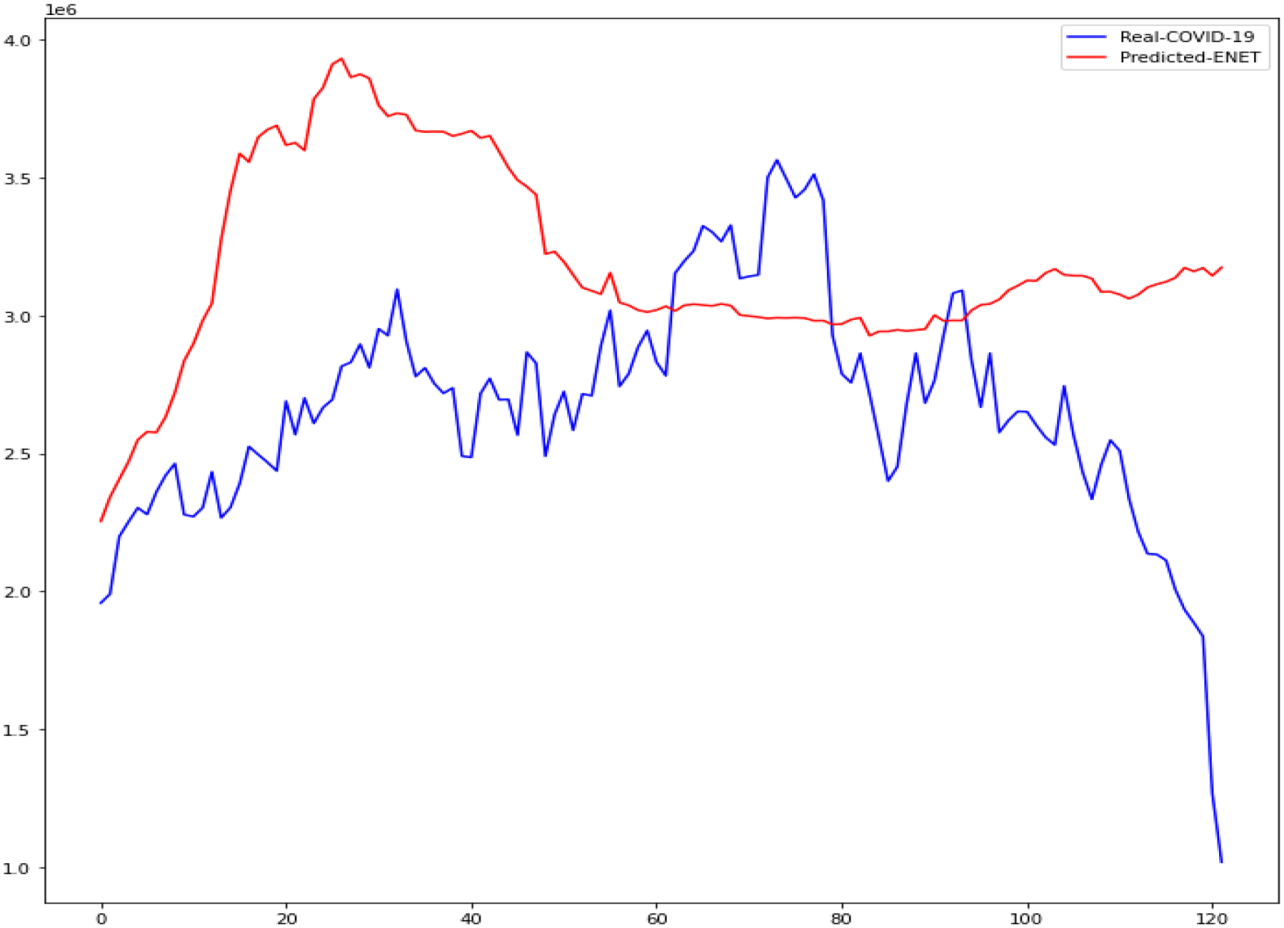
COVID-19 Vaccination of Real and Predicted (ENET)

**Fig. 14:**
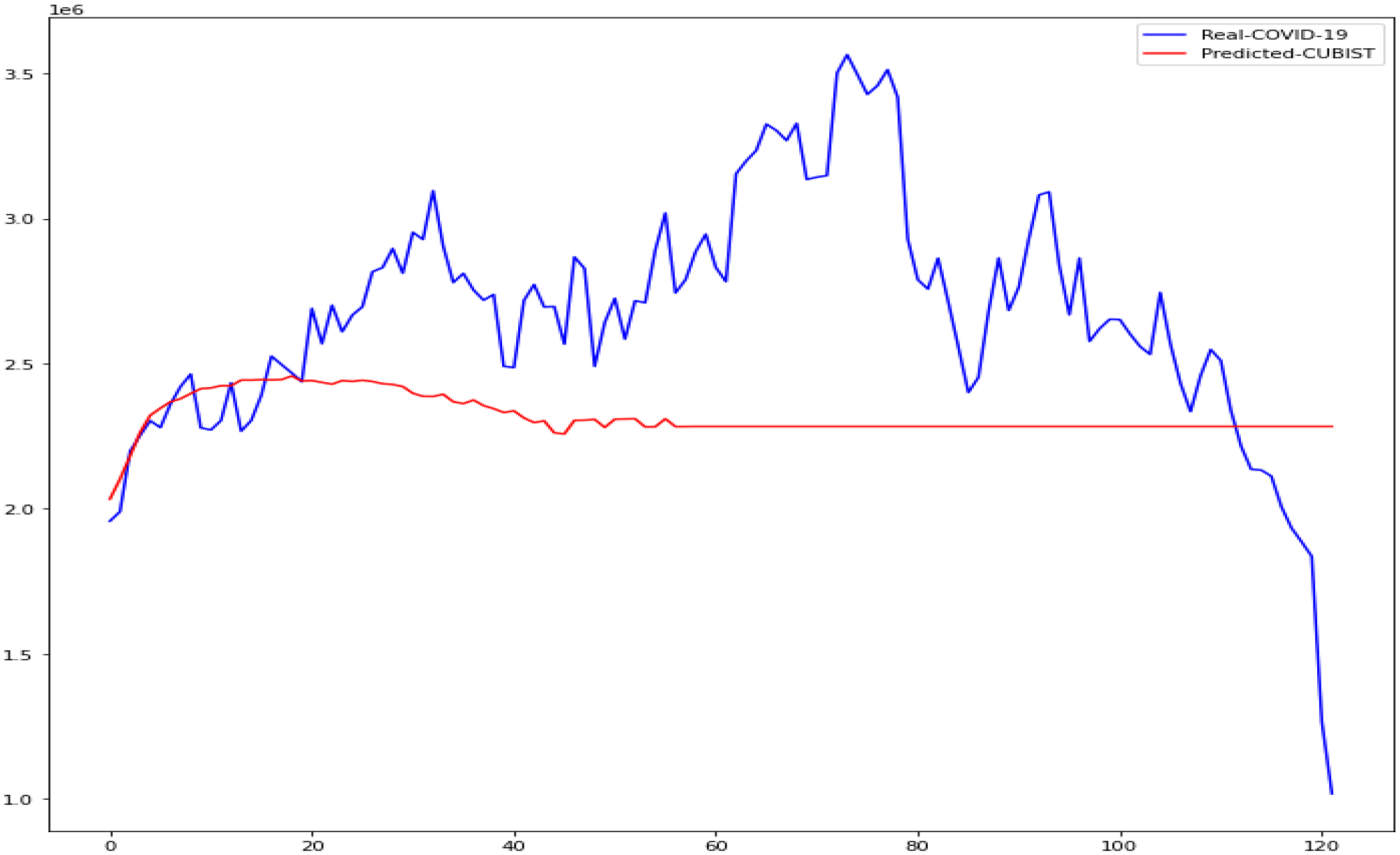
COVID-19 Vaccination of Real and Predicted (CUBIST)

**Fig. 15:**
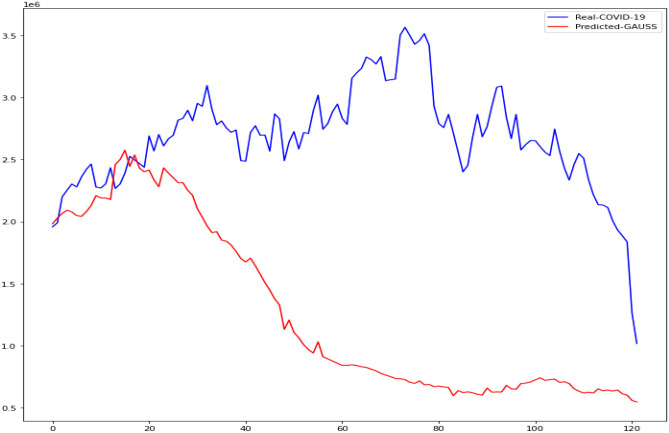
COVID-19 Vaccination of Real and Predicted (GAUSS)

**Fig. 16:**
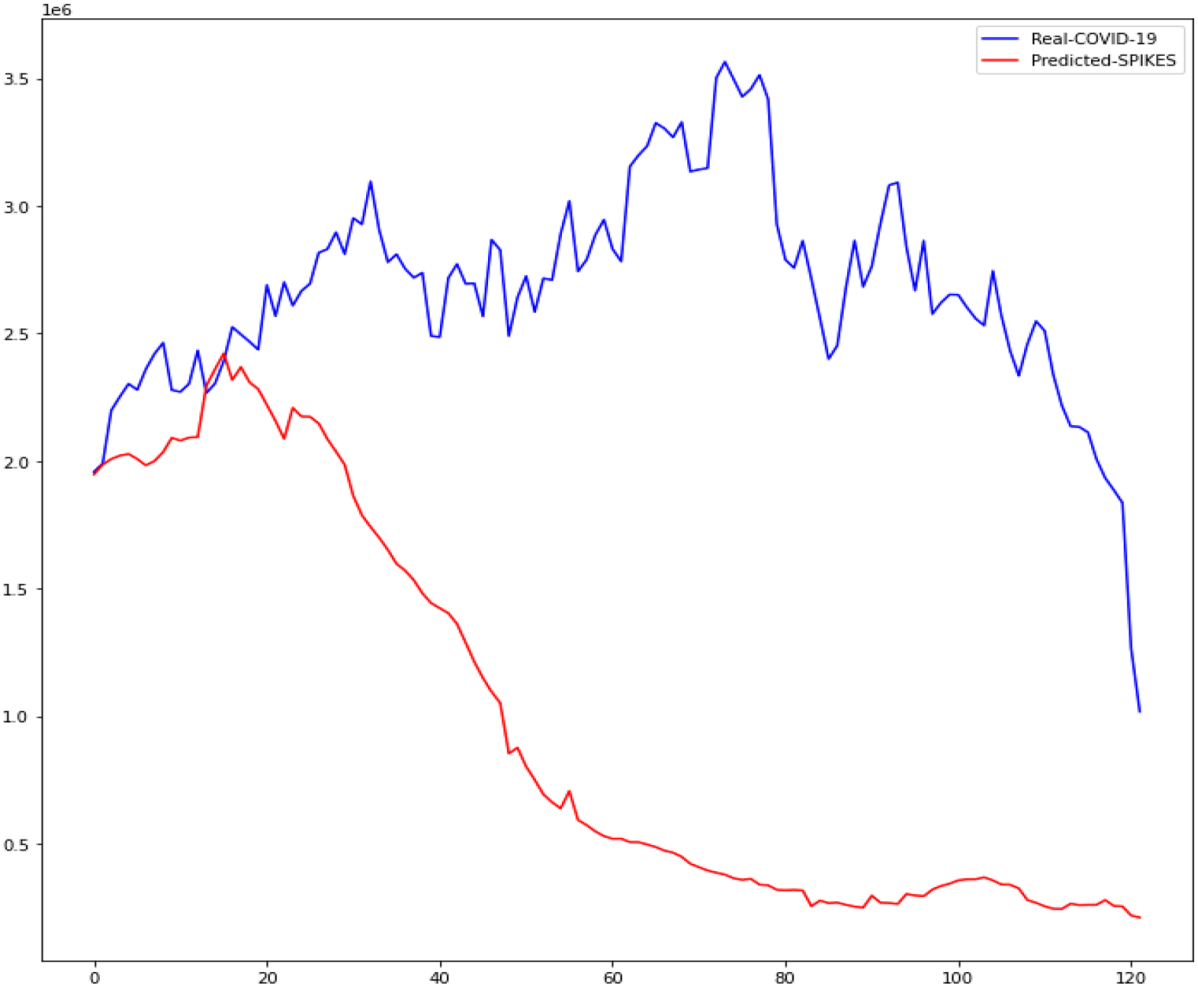
COVID-19 Vaccination of Real and Predicted (SPIKES)

In this research, the outcomes of machine learning approaches such as CUBIST, Spikes and Slab (SPIKES), Gaussian Process (GAUSS), and Elastic Net (ENET) were studied. The four machine learning algorithms used in this study were compared to determine which was more accurate in predicting daily COVID-19 immunization. The Kaggle database was used to get the COVID-19 dataset. We demonstrate the accuracy of these methods in predicting daily COVID-19 vaccination using Mean Absolute Scaled Error (MASE), Relative Absolute Error (RAE), and Mean Squared Log Error (MSLE). Figures 13, 14, 15, 16 is the result of both real and predicted COVID-19 vaccination of all the four machine learning considered in this paper.

MASE, RAE, and MSLE are among the performance measures shown in Table 4. CUBIST surpasses the other three algorithms by attaining lower error rates, implying that the methods are more precise than any other forecast. According to the data, CUBIST can predict daily COVID-19 immunization.

**Table 4:** Performance Measures of ENET, CUBIST, GAUSS AND SPIKES

**Continent**	**Models**	**MASE**	**MSLE**	**RAE**
Africa	ENET	16.7807	0.5399	1.8707
	CUBIST	10.4519	0.4294	1.1651
	GAUSS	8.4411	0.2062	0.9410
	SPIKES	8.4079	0.2048	0.9373
Asia	ENET	24.4637	0.4161	4.8801
	CUBIST	9.7368	0.1678	1.9423
	GAUSS	17.0896	0.2579	3.4091
	SPIKES	23.9455	0.4041	4.7768
Europe	ENET	43.2658	0.6320	2.5360
	CUBIST	11.8689	0.1783	0.6957
	GAUSS	9.1852	0.0865	0.5384
	SPIKES	8.3149	0.0564	0.4873
North America	ENET	30.9528	0.6220	10.1623
	CUBIST	2.8901	0.0178	0.9488
	GAUSS	84.0509	-	27.5954
	SPIKES	76.2009	-	25.0181
Oceania	ENET	39.0103	0.9809	1.9631
	CUBIST	13.2169	0.0990	0.6651
	GAUSS	24.1195	0.2430	1.2137
	SPIKES	21.3783	0.1696	1.0758
South America	ENET	5.5114	0.0724	2.0113
	CUBIST	3.9510	0.0473	1.4419
	GAUSS	12.7782	1.0958	4.6633
	SPIKES	15.2499	2.5426	5.5653

## Conclusions

A study was carried out for the prediction of daily COVID-19 to reduce disease propagation. The study presented and used four well-known machine learning models for daily COVID-19 prediction: CUBIST, Gaussian Process (GAUSS), Elastic Net (ENET), and Spikes and Slab (SPIKES). According to the findings of the study, CUBIST have the ability to predict daily COVID-19 immunization. As the findings demonstrate, selecting the best successful model for this prediction requires a combination of performance indicators such as Relative Absolute Error (RAE), Mean Squared Log Error (MSLE), and Mean Absolute Scaled Error (MASE). CUBIST can predict daily COVID-19 immunization in Asia with 9.7368 (MASE), North America with 2.8901 (MASE), Oceania with 13.2169 (MASE) and South America with 3.9510, respectively. Furthermore, findings showed that different nations in Europe begin immunizing their citizens earlier than other continents.

However, the limitation of this research is that obtaining COVID-19 datasets of those who are fully vaccinated is extremely difficult due to the fact that social media is awash with posts denigrating the vaccine hesitant. This has resulted in many people being unwilling to receive COVID-19 vaccination. The consequence is something of a social media cultural war, with many online commentators suggesting that vaccine skeptics are altering their minds, yet even a delay is considered a hazard to health because viral diseases spread swiftly. In the future, we will examine various machine learning techniques to assess daily COVID-19 vaccination.

## Data Availability

The data is available at https://www.kaggle.com/gpreda/covid-world-vaccination-progress

## References

[1] World Health Organization. Coronavirus. Available online: https://www.who.int/emergencies/diseases/novel-coronavirus-2019. Accessed 20 Jan 2022.

[2] Adans-Dester CP, Bamberg S, Bertacchi FP, Caulfield B, Chappie K, Demarchi D, Bonato P (2020). Can mHealth technology help mitigate the effects of the COVID-19 pandemic?. IEEE Open J Eng Med Biol.

[3] Swayamsiddha S, Mohanty C. Application of cognitive Internet of Medical Things for COVID-19 pandemic. Diabetes & Metabolic Syndrome: Clin Res Rev. 2020. 10.1016/j.dsx.2020.06.014 PMC7287427 32570016

[4] Shinan-Altman S, Levkovich I, Tavori G (2020). Healthcare utilization among breast cancer patients during the COVID-19 outbreak. Palliat Support Care.

[5] Achieving 70% COVID-19 Immunization Coverage by Mid-2022. World Health Organization. Available at https://www.who.int/news/item/23-12-2021-achieving-70-covid-19-immunization-coverage-by-mid-2022. Accessed 30 Sept 2022.

[6] Eisenstadt M, Ramachandran M, Chowdhury N, Third A, Domingue J (2020). COVID-19 antibody test/vaccination certification: there's an app for that. IEEE Open J Eng Med Biol.

[7] Arowolo MO, Ogundokun RO, Misra S, Agboola BD, Gupta B. Machine learning-based IoT system for COVID-19 epidemics. Computing. 2022;1–17.

[8] Oyewola DO, Dada EG, Al-Mustapha KA, Fadele AA, Joseph SB, Ibrahim A. Predicting Transmission Rate of Coronavirus (COVID-19) Pandemic Using Machine Learning Techniques. In: Kose U, Watada J, Deperlioglu O, Marmolejo Saucedo JA (eds) Computational Intelligence for COVID-19 and Future Pandemics. Disruptive Technologies and Digital Transformations for Society 5.0. Springer, Singapore, 2022. 10.1007/978-981-16-3783-4_3.

[9] Oyewola DO, Al-Mustapha KA, Ibrahim A, Dada EG. High-Performing Machine Learning Algorithms for Predicting the Spread of COVID-19. In: Faghih N, Forouharfar A (eds) Socioeconomic Dynamics of the COVID-19 Crisis. Contributions to Economics. Springer, Cham., Switzerland, 2022. 10.1007/978-3-030-89996-7_17.

[10] Passarelli-Araujo H, Passarelli-Araujo H, Urbano MR, Pescim RR. Machine learning and comorbidity network analysis for hospitalized patients with COVID-19 in a city in Southern Brazil. Smart Health. 2022;100323. 10.1016/j.smhl.2022.100323 PMC9485420 36159078

[11] Oyewola DO, Dada EG, Misra S, Damaševičius R. A Novel Data Augmentation Convolutional Neural Network for Detecting Malaria Parasite in Blood Smear Images, Appl Artif Intell (Accepted for publication). 2022.

[12] Hasan M, Bath P, Marincowitz C, Sutton L, Pilbery R, Hopfgartner F, Goodacre S. Pre-Hospital Prediction of Adverse Outcomes in Patients with Suspected COVID-19: Development, Application and Comparison of Machine Learning and Deep Learning Methods. Comput Biol Med. 2022. 10.1016/j.compbiomed.2022.106024. 10.1016/j.compbiomed.2022.106024 PMC9420071 36327887

[13] Afrash MR, Kazemi-Arpanahi H, Shanbehzadeh M, Nopour R, Mirbagheri E. Predicting hospital readmission risk in patients with COVID-19: a machine learning approach. Inform Med Unlocked. 2022;30:100908. 10.1016/j.imu.2022.100908 PMC8901230 35280933

[14] Dada EG, Bassi JS, Chiroma H, Adetunmbi AO, Ajibuwa OE (2019). Machine learning for email spam filtering: review, approaches and open research problems. Heliyon.

[15] Oyewola DO, Dada EG, Misra S, Damaševičius R. Detecting Cassava Mosaic Disease Using A Deep Residual Convolutional Neural Network With Distinct Block Processing. PeerJ Comput. Sci., USA, 2021;7:e352. 10.7717/peerj-cs.352. 10.7717/peerj-cs.352 PMC7959600 33817002

[16] Ibrahim I, Abdulazeez A (2021). The Role of Machine Learning Algorithms for Diagnosing Diseases. J Appl Sci Technol Trends.

[17] Nithya B, Ilango V. Predictive analytics in health care using machine learning tools and techniques. In 2017 International Conference on Intelligent Computing and Control Systems (ICICCS) (pp. 492–499). IEEE. 2017.

[18] Velu A, Reddy R, Sharma P. Impact of Covid Vaccination on the Globe using data analytics. In Swarm Intelligence and Machine Learning (pp. 21–33). CRC Press. 2021.

[19] To QG, To KG, Huynh VAN, Nguyen NT, Ngo DT, Alley SJ, Vandelanotte C (2021). Applying machine learning to identify anti-vaccination tweets during the COVID-19 pandemic. Int J Environ Res Public Health.

[20] Magazzino C, Mele M, Coccia M (2022). A machine learning algorithm to analyse the effects of vaccination on COVID-19 mortality. Epidemiol Infect.

[21] Wedlund L, Kvedar J (2021). New machine learning model predicts who may benefit most from COVID-19 vaccination. NPJ Digit Med.

[22] Kim M (2021). Prediction of COVID-19 Confirmed Cases after Vaccination: Based on Statistical and Deep Learning Models. SciMedicine J.

[23] Cheong Q, Au-Yeung M, Quon S, Concepcion K, Kong JD (2021). Predictive Modeling of Vaccination Uptake in US Counties: A Machine Learning-Based Approach. J Med Internet Res.

[24] Abdulkareem NM, Abdulazeez AM, Zeebaree DQ, Hasan DA (2021). COVID-19 world vaccination progress using machine learning classification algorithms. Qubahan Acad J.

[25] Fernandes N, Costa D, Costa D, Keating J, Arantes J (2021). Predicting COVID-19 vaccination intention: the determinants of vaccine hesitancy. Vaccines.

[26] Zaidi SAJ, Tariq S, Belhaouari SB (2021). Future Prediction of COVID-19 Vaccine Trends Using a Voting Classifier. Data.

[27] Davahli MR, Karwowski W, Fiok K. Optimizing COVID-19 vaccine distribution across the United States using deterministic and stochastic recurrent neural networks. PLoS One. 2021;16(7):e0253925. 10.1371/journal.pone.0253925 PMC8259963 34228740

[28] Liu W, Li Q. An Efficient Elastic Net with Regression Coefficients Method for Variable Selection of Spectrum Data. PLoS One. 2017;12(2):e0171122. 10.1371/journal.pone.0171122. 10.1371/journal.pone.0171122 PMC5289531 28152003

[29] Waldmann P, Meszaros G, Gredler B, Fuerst C, Solkner J (2013). Evaluation of the lasso and the elastic net in genome-wide association studies. Front Genet.

[30] Zhou J, Li E, Wei H, Li C, Qiao Q, Armaghani DJ (2019). Random forests and cubist algorithms for predicting shear strengths of rockfill materials. Appl Sci.

[31] Wang J. An intuitive tutorial to Gaussian processes regression. 2020. arXiv preprint arXiv:2009.10862.

[32] Cheng L, Ramchandran S, Vatanen T, Lietzén N, Lahesmaa R, Vehtari A, Lähdesmäki H (2019). An additive Gaussian process regression model for interpretable non-parametric analysis of longitudinal data. Nat Commun.

[33] Mitchell TJ, Beauchamp JJ (1988). Bayesian Variable Selection in Linear Regression. J Am Stat Assoc.

[34] Madigan D, Raftery AE (1994). Model Selection and Accounting in Graphical Models for Model Uncertainty Using Occam’s Window. J Am Stat Assoc.

[35] George EI, McCulloch RE. Approaches for Bayesian variable selection. Statistica sinica. 1997;339–373.

[36] Ishwaran H, Rao JS (2005). Spike and slab variable selection: frequentist and Bayesian strategies. Ann Stat.

[37] Kaggle. COVID-19 World Vaccination Progress. Daily and Total Vaccination for COVID-19 in the World from Our World in Data. 2022. Available at https://www.kaggle.com/gpreda/covid-world-vaccination-progress. Accessed 5 Jan 2022.

